# Distribution and Viability of Fetal and Adult Human Bone Marrow Stromal Cells in a Biaxial Rotating Vessel Bioreactor after Seeding on Polymeric 3D Additive Manufactured Scaffolds

**DOI:** 10.3389/fbioe.2015.00169

**Published:** 2015-10-23

**Authors:** Anne M. Leferink, Yhee-Cheng Chng, Clemens A. van Blitterswijk, Lorenzo Moroni

**Affiliations:** ^1^Department of Tissue Regeneration, MIRA Institute, University of Twente, Enschede, Netherlands; ^2^Department of Complex Tissue Regeneration, Faculty of Health, Medicine and Life Sciences, Maastricht University, Maastricht, Netherlands; ^3^Quintech Life Sciences Pte Ltd., Singapore, Singapore

**Keywords:** bone marrow stromal cells, scaffolds, biaxial rotating bioreactor, perfusion-flow bioreactor, cellular distribution

## Abstract

One of the conventional approaches in tissue engineering is the use of scaffolds in combination with cells to obtain mechanically stable tissue constructs *in vitro* prior to implantation. Additive manufacturing by fused deposition modeling is a widely used technique to produce porous scaffolds with defined pore network, geometry, and therewith defined mechanical properties. Bone marrow-derived mesenchymal stromal cells (MSCs) are promising candidates for tissue engineering-based cell therapies due to their multipotent character. One of the hurdles to overcome when combining additive manufactured scaffolds with MSCs is the resulting heterogeneous cell distribution and limited cell proliferation capacity. In this study, we show that the use of a biaxial rotating bioreactor, after static culture of human fetal MSCs (hfMSCs) seeded on synthetic polymeric scaffolds, improved the homogeneity of cell and extracellular matrix distribution and increased the total cell number. Furthermore, we show that the relative mRNA expression levels of indicators for stemness and differentiation are not significantly changed upon this bioreactor culture, whereas static culture shows variations of several indicators for stemness and differentiation. The biaxial rotating bioreactor presented here offers a homogeneous distribution of hfMSCs, enabling studies on MSCs fate in additive manufactured scaffolds without inducing undesired differentiation.

## Introduction

The field of tissue engineering aims at applying the fundamentals of cell biology and materials engineering to construct replacements for damaged, diseased, or lost tissue (Langer and Vacanti, 1993). One of the conventional approaches is based on three-dimensional (3D) mechanically stable scaffolds in combination with multipotent cell types. Scaffolds with a highly defined geometry, porosity, and tailored mechanical properties can be obtained by additive manufacturing (Moroni et al., 2005). These scaffolds provide the necessary support for cells to attach, proliferate, and differentiate, and define the overall shape of the tissue engineered transplant. Several researchers already showed successful application of such scaffolds in, for example, bone and cartilage tissue engineering *in vivo* (Woodfield et al., 2004; Kim et al., 2012; Reichert et al., 2012). Woodfield et al. found a rapid attachment and a homogenous distribution of both bovine and human chondrocytes on poly (ethylene oxide terephthalate)-co-poly (butylene terephthalate) (PEOT/PBT) based scaffolds after spinner flask culture *in vitro*. Subsequently, scaffolds seeded with bovine chondrocytes were implanted subcutaneously in mice for 21 days and formed cartilaginous tissue *in vivo* as demonstrated by the presence of articular extracellular matrix (ECM) components (Woodfield et al., 2004). Despite successes in regenerating tissues with additive manufactured scaffolds optionally combined with different bioreactors, the highly organized open structure of such scaffolds poses still challenges in homogenously distributing cells and controlling their proliferation and differentiation capacities. This is even more important when mesenchymal stromal cell (MSCs) are used, as their capacity to adhere and be homogeneously distributed in 3D scaffolds has shown to be more demanding (Griffon et al., 2011).

To gain control over cell seeding efficiency, distribution and fate on additive manufactured 3D scaffolds *in vitro* prior to implantation or as a study model, several hurdles have to be overcome. First, the cell seeding process has to be optimized per scaffold geometry, scaffold material, and cell type to achieve proper cell attachment and distribution throughout the construct (Sobral et al., 2011). Several studies showed the influence of scaffold geometry or culture conditions in cell and tissue distribution after *in vitro* culture (Wang et al., 2005; Leferink et al., 2013). Papadimitropoulos et al. (2013) introduced a collagen-network in porous PCL–TCP scaffolds, which resulted in a 2.5-fold increase in MSCs seeding efficiency under perfusion flow compared to the bare PCL–TCP scaffolds. Despite an increased cell seeding efficiency in the presence of a collagen-network, no obvious qualitative differences were found after 19 days of culture among the experimental groups with respect to cell viability and distribution, and ECM formation. Although the incorporation of a collagenous matrix in additive manufactured scaffolds seemed to have a beneficial effect on cell seeding efficiency, control over cell fate remains to be further elucidated.

A second hurdle to overcome is the supply of oxygen and nutrients as well as the clearance of metabolic products which showed to become critically limiting for cells cultured under static conditions (Schantz et al., 2012). Bioreactor systems, based on convection, such as rotating vessels and stirrer flasks, or based on perfusion, such as a directional flow-through bioreactor, are used to overcome these mass transfer limitations (Grayson et al., 2011). These systems do not only enhance nutrient and waste product exchange but can also exert mechanical stimuli on the cells to proliferate, migrate, or differentiate (Martin et al., 2004; Grayson et al., 2011). Although most of these commercially available rotating vessel bioreactors are uniaxial in design, Singh et al. (2005) have shown with *in silico* simulations that a biaxial design, which rotates simultaneously in two independent orthogonal axes, resulted in improved fluidics over an uniaxial design. Therefore, we investigated the use of a biaxial bioreactor system for the *in vitro* culture of highly porous PEOT/PBT scaffolds seeded with human fetal MSCs (hfMSCs) or human adult MSCs (haMSCs).

Previous work showed the differentiation potential of hfMSCs into the adipogenic (Jo et al., 2008), osteogenic (Guillot et al., 2008; Abarrategi et al., 2012; Brady et al., 2014), and chondrogenic (Abarrategi et al., 2012; van Gool et al., 2012) lineages and maintenance of telomerase activity during *in vitro* monolayer culture (Jo et al., 2008). Also for haMSCs, multipotency is traditionally shown by studying the differentiation capacity into the adipogenic (Pittenger et al., 1999), chondrogenic (Mackay et al., 1998; Pittenger et al., 1999), and osteogenic (Pittenger et al., 1999) lineages in the presence of soluble factors in monolayer or pellet culture *in vitro*. Yet, the differentiation of haMSCs into multiple other lineages, such as the neurogenic (Zaim et al., 2012), endothelial (Janeczek Portalska et al., 2012), and myogenic lineages (Muguruma et al., 2003; Bossolasco et al., 2004), has been reported as well. The multipotent differentiation capacity of haMSCs is donor and age dependent and decreases upon increasing population doublings *in vitro* (Zaim et al., 2012). Zhang et al. (2009a) reported a superior proliferative and osteogenic differentiation capacity of hfMSCs over haMSCs after comparative studies *in vitro* in static monolayer culture. In addition, both hfMSCs and haMSCs showed osteogenic differentiation on a composite bioactive PCL–TCP scaffold in static culture *in vitro* and ectopic bone formation in the scaffold after implantation *in vivo*. Yet, osteogenic differentiation of hfMSCs was found superior to haMSCs.

We hypothesize that by the introduction of a biaxial rotating bioreactor, higher cell numbers and a more homogeneous distribution of cells throughout 3D scaffolds could be achieved compared to static culture in a well plate. It is well-known that shear forces due to medium flow can affect cell fate. Therefore, in this study, the effect of bioreactor culture on cell and ECM distribution as well as on cellular phenotype was assessed for both hfMSCs and haMSCs cultured on scaffolds in two different bioreactor systems and compared to the results from static culture on scaffolds in a well plate. In our study, we chose PEOT/PBT as a biomaterial to fabricate 3D additive manufactured scaffolds. This family of copolymers has shown successful applications in tissue regeneration applications (Beumer et al., 1994; Claase et al., 2007; Moroni et al., 2008), due to the flexibility to change their physico-chemical and mechanical properties (Deschamps et al., 2002). These results in the possibility to control cell adhesion, morphology, and ultimately phenotype at the interface with 3D scaffolds made of these biomaterials, which is of particular interest for stem cell-driven tissue regeneration strategies.

## Materials and Methods

### Isolation and Culture of hfMSCs and haMSCs

Bone marrow-derived hfMSCs were isolated as described before, from 17 weeks and 1-day-old fetuses after clinically indicated termination of pregnancy (Chan et al., 2007; Zhang et al., 2009b). Pregnant women gave separate written consent for the clinical procedure and for the use of fetal tissue for research purposes. Briefly, fetal bone marrow cells were retrieved by flushing the bone marrow cells out of the humeri and femurs into Dulbecco’s modified eagle’s medium (DMEM, Sigma, USA) with Glutamax (GIBCO, USA) supplemented with 10% heat-inactivated fetal bovine serum (FBS, Hyclone, USA), 50 U/mL penicillin, and 50 μg/mL streptomycin (GIBCO, USA), which will be referred to as fMSC medium. Medium was refreshed twice per week and cells were used for further subculturing or cryopreservation on reaching near confluence.

Bone marrow-derived haMSCs (donor 1 female, 77 years old; donor 2 female, 55 years old) were isolated and proliferated, as described previously (de Bruijn et al., 1999). Bone marrow aspirates were obtained from patients who had given written informed consent. Briefly, aspirates were cultured in minimal essential medium (alpha-MEM; Life Technologies, USA) supplemented with 10% heat-inactivated fetal bovine serum (FBS; Lonza, USA), 0.2 mM l-Ascorbic acid 2-phosphate magnesium salt (ASAP, Sigma-Aldrich, the Netherlands), 2 mM l-glutamine (l-glut, Invitrogen, the Netherlands), 100 U/mL penicillin (Life Technologies, USA), 100 μg/mL streptomycin (Life Technologies, USA), and 1 ng/mL basic fibroblast growth factor (bFGF; Instruchemie, The Netherlands), which will be referred to as proliferation medium. Cells were cultured at 37°C in a humidified atmosphere with 5% CO_2_. Medium was refreshed twice per week and cells were used for further subculturing or cryopreservation on reaching near confluence.

### Fabrication of PEOT/PBT Scaffolds

Scaffold were fabricated of PolyActive™ 300/55/45 (PolyVation, the Netherlands), a block copolymer is composed of poly(ethylene oxide terephthalate) (PEOT) and poly(butylene terephthalate) (PBT) with a weight ratio of 55:45 for the two components, respectively, and a molecular weight of the starting poly(ethylene glycol) (PEG) segments of 300 Da used in the co-polymerization process (Deschamps et al., 2002). Fused deposition modeling was used with a bioscaffolder (SysENG, Germany) to fabricate 3D cylindrical scaffolds as described before (Moroni et al., 2005), with a diameter of 8 mm and a height of 3 mm. For the additive manufacturing process, the fiber spacing was set to 1000 μm, the layer thickness to 150 μm, and a needle with an internal diameter of approximately 250 μm was used, which resulted in fiber diameters of approximately 200 μm. Scaffolds were treated in Argon plasma for 30 min with a pressure of 0.1–0.2 mBar and a power of 30 W. Sterilization of all scaffolds was performed in 70% ethanol twice for 30 min, subsequently washed in PBS first for 5 min and additionally twice for another 30 min each time, and finally incubated in culture medium overnight prior to cell culture.

### Cell Seeding on PEOT/PBT Scaffolds

For studies with hfMSCs, scaffolds were transferred from a tube with culture medium to a non-treated 24-well plate (Greiner bio-one, Germany). Passage 3 hfMSCs were harvested from monolayer expansion, and seeded on the scaffolds with a density of 750,000 cells in 30 μL of fMSC medium. After 15 min of incubation, the scaffolds were flipped and allowed to incubate for another 45 min before 10 μL of fMSC medium was added. Subsequently, for the next 5 h, 10 μL of proliferation medium was added for each seeded scaffolds at an interval of 75 min for each addition. Scaffolds were then transferred to a non-treated 24-well plate (Greiner bio-one, Germany) with 750 μL of proliferation medium and cultured for 3 days either in static culture conditions or in a biaxial rotating vessel bioreactor for which the culture parameters are specified in the next section.

For studies with haMSCs, scaffolds were transferred from a tube with culture medium to a non-treated 24-well plate (NUNC, Fisher Scientific, the Netherlands). Passage 3 haMSCs were harvested from monolayer expansion, and seeded on the scaffolds with a density of 750,000 cells in 100 μL of proliferation medium. After 1.5 h incubation, the medium was filled up to 1 mL and cell culture was continued for 3 days in static culture condition before transferring the samples to a new culture well, to the biaxial bioreactor system, or to a bioreactor perfusion culture. The culture parameters in the bioreactors are specified in the next section.

In the results section, the legends of the figures refer to the total culture time, including the static incubation period on the scaffolds placed in a well plate (in most figures this period was 3 days, unless specified differently).

### Bioreactor Culture

**Figure 1:**
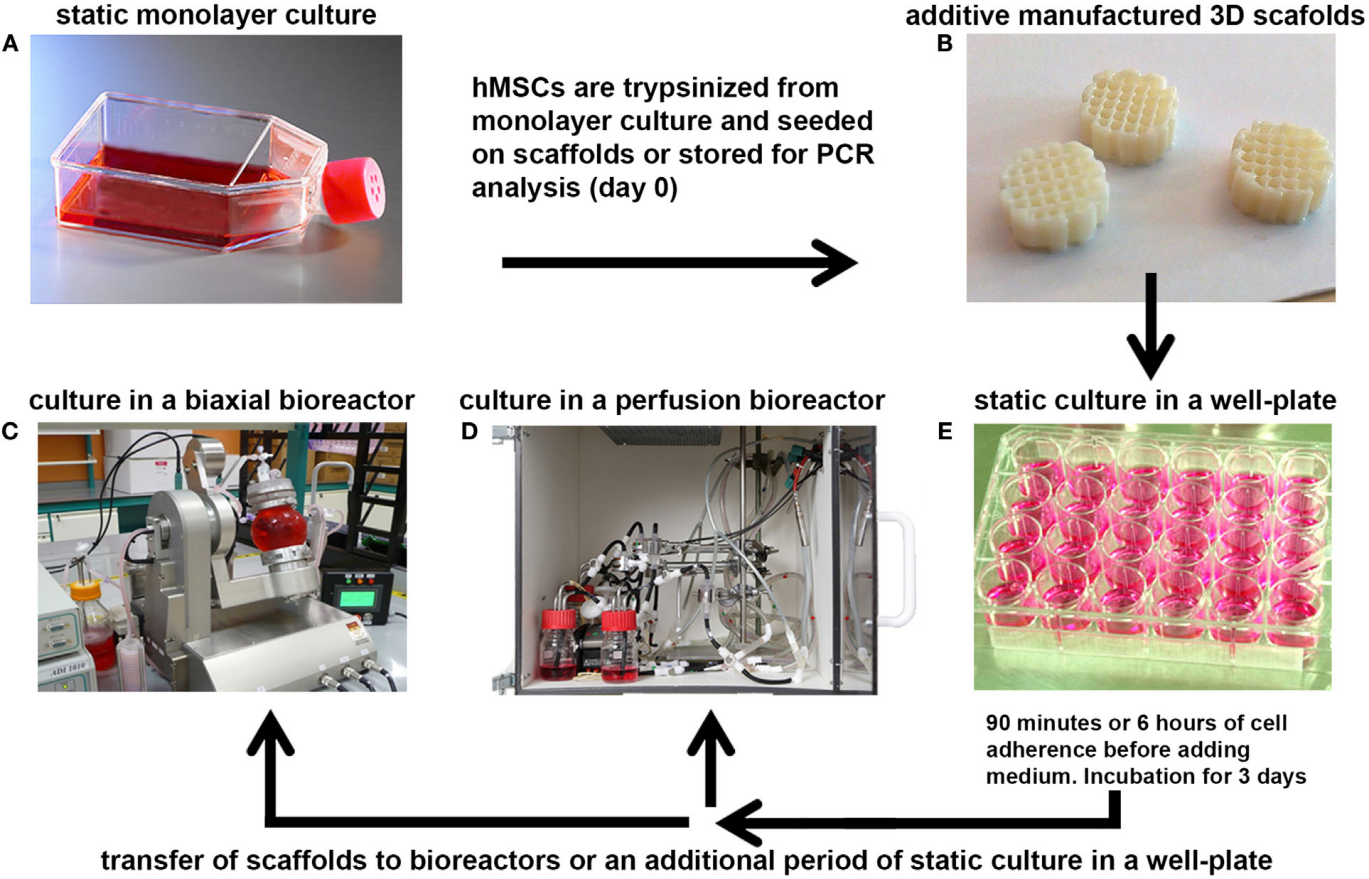
**A schematic representation of the bioreactor set-ups**. **(A)** hMSCs were cultured at 37°C in a humidified atmosphere with 5% CO2 in culture flasks to allow them to proliferate. After expansion, the cells were trypsinized from monolayer culture and seeded onto 3D additive manufactured scaffolds **(B)**, or stored for analysis of the basal gene expression levels (day 0). **(E)** Seeded scaffolds were statically incubated for 90 min or 6 h in a 24-well plate to allow the hMSCs to adhere to the 3D scaffold before filling the well with culture medium. After several days of incubation, the scaffolds were transferred to a small press-fit chamber in the perfusion bioreactor **(D)** or in the vessel of the biaxial rotating bioreactor **(C)** in which culture was prolonged for several days before analyses.

After static culture, some of the scaffolds were transferred to the biaxial bioreactor system or the chamber of the perfusion bioreactor, whereas a control group was maintained under static culture conditions (Figure 1). The perfusion bioreactor consisted of a small chamber in which the scaffold was press-fitted to ensure the medium to be flown through the longitudinal pores of the scaffold. The set-up of the perfusion bioreactor was constructed, as described before (Janssen et al., 2006). Medium was ran with a flow speed of 0.1 mL/min in a home-made incubator at 37°C with a controlled flow of mixed gas containing 5% CO_2_ and 21% O_2_.

The biaxial bioreactor system consisted of a cylindrical vessel for culture (volume 40 mL), in which the cellular-scaffold constructs are mounted to the lid of the bioreactor by pins, and a medium reservoir to ensure continuous replacement of the medium in the culture vessel. Gaseous exchange was enabled through a special membrane incorporated into the medium reservoir. The bioreactor was ran with a medium flow speed of 1 rpm (corresponding to 1.5 mL/min), a biaxial rotation with an arm rotational speed of 2 rpm, and a chamber rotation speed of 3 rpm at 37°C in a humidified atmosphere with 5% CO_2_.

Proliferation medium was used to fill the 40 mL medium reservoir of the perfusion bioreactor containing 1 scaffold seeded with haMSCs per circuitry. Proliferation medium was used to fill the 40 mL bioreactor chamber and the 300 mL reservoir of the biaxial bioreactor containing 4 scaffolds seeded with haMSCs. fMSC medium was used to fill the 500 mL bioreactor chamber and the 300 mL reservoir of the biaxial bioreactor containing 12 scaffolds seeded with hfMSCs. As a control, static culture was continued on the scaffolds that were transferred to a new well in a non-treated 24-well plate.

### Viability Staining

Cell viability was assessed by 4 μg/mL fluorescein diacetate (FDA, Sigma-Aldrich, the Netherlands) and 40 μg/mL propidium iodide (PI, Sigma-Aldrich, the Netherlands) staining, where FDA stains viable cells green, and PI stains necrotic and apoptotic cell nuclei red. Scaffolds were cut in half, stained with FDA and PI, as previously described (Zhang et al., 2009a), and viewed under a confocal laser microscope (Olympus, FV1000Fluoview, Japan). Cellular scaffolds were examined in both planar view and cross-sectional view after 3 and 9 days of culture.

### DNA Assay

The total DNA from scaffolds cultured with hfMSCs was extracted from each scaffold by incubating the constructs in 0.4 mL of enzymatic cocktail [consisting of 0.1% collagenase A (Roche, Switzerland) with 0.1% Trypsin mixed in PBS] at 37°C for 2 h, with vortex every 30 min followed by three cycles of freeze and thaw. For haMSCs, after culture all scaffolds were washed gently in PBS, dried by aspirating the PBS, cut in pieces and stored at −80°C for at least 24 h. After thawing, the constructs were digested for 16 h at 56°C with 1 mg/mL proteinase K (Sigma-Aldrich, the Netherlands) in Tris/EDTA buffer (pH 7.6). This solution contained 18.5 μg/mL iodoacetamine (Sigma-Aldrich, the Netherlands) and 1 μg/mL Pepstatin A (Sigma-Aldrich, the Netherlands). Quantification of total DNA was done using the CyQuant^®^ DNA assay (Molecular Probes, Fisher Scientific, the Netherlands) and a spectrophotometer (excitation 480 nm, emission 520 nm) (Victor 3, Perkin Elmer, the Netherlands).

### Hydroxyproline Assay

Hydroxyproline colorimetric assay kit (Biovision, USA) was performed according to the manufacturer’s protocol to determine the total collagen amount on the same samples as used for DNA quantification. In brief, from the digested sample, 30 μL was transferred to a Teflon^®^ capped glass bottle. Another 30 μL of concentrated hydrochloric acid (HCl, 12M, Sigma-Aldrich, the Netherlands) was added and samples were hydrolyzed at 120°C for 3 h. The complete supernatant was transferred to a 96-well plate and left to evaporate at 60°C. Subsequently, 100 μL of chloramine T/Oxidation buffer mix (Biovision, USA) was added to each well and incubated at room temperature for 5 min. Finally, 100 μL of DMAB Reagent (Biovision, USA) was added to each well including the hydroxyproline standard and incubated for 90 min at 60°C. A micro plate reader (Multiskan GO, Thermo Fisher, the Netherlands) was used to determine the absorbance at 560 nm.

### Gene Expression Analysis of hfMSCs and haMSCs

For gene expression analysis of hfMSCs, samples were seeded (*n* = 4 for static cultures; *n* = 5 for the biaxial bioreactor system) at different time points. After culture, medium was aspirated from the scaffolds, the samples were transferred to 2 mL Eppendorf tubes and stored at −80°C. Prior to RNA isolation from the samples by using a Bioke RNA II nucleospin RNA isolation kit (Macherey-Nagel, Germany), first 500 μL of TRIzol^®^ (Invitrogen, the Netherlands) was added. The scaffolds were disrupted by crushing with RNA isolation pestles (Kimble Kontes, Fisher Scientific, the Netherlands). Samples for basal gene expression analysis, referred to as T25 day 0, were retrieved from two-dimensional (2D) culture in T25 tissue culture flasks (NUNC, Fisher Scientific, the Netherlands) by adding 500 μL of TRIzol^®^ (Invitrogen, the Netherlands) upon reaching 70% confluence. Subsequently, the cell/TRIzol^®^ suspension was transferred to an Eppendorf tube and 200 μL of CHCl_3_ was added to each sample and mixed by vigorously shaking the tubes. The TRIzol^®^/CHCl_3_ mixture was centrifuged at 12,000 × g for 15 min at 4°C. The aqueous phase was transferred to a new Eppendorf tube and mixed 1:1 with 70% ethanol. The mixture was transferred to filter columns from the kit and the RNA isolation was continued following the manufacturer’s protocol. RNA concentrations and purity were determined by using an ND1000 spectrophotometer (Nanodrop Technologies, USA). The cDNA was synthesized from 630 ng of RNA, using iScript™ (BIO-RAD, Bio-rad Laboratories, the Netherlands) according to the manufacturer’s protocol. Quantitative polymerase chain reaction (qPCR) was performed on cDNA samples by using the iQ SYBR^®^ Green Supermix (Bio-Rad, the Netherlands) on the primers as listed in Table 1. PCR reactions were carried out on the MyiQ2 Two-Color Real-Time PCR Detection System (Bio-Rad, the Netherlands) under the following conditions: cDNA was denatured for 10 min at 95°C, followed by 40 cycles, consisting of 15 s at 95°C, 30 s at 60°C, and 30 s at 72°C. For each reaction, a melting curve was generated to test primer dimer formation and non-specific priming. The cycle threshold (Ct) values were determined with the Bio-RadiQ5 optical system software, in which a threshold value was set for the fluorescent signal at the lower log-linear part above the baseline. Ct values were normalized to the B2M housekeeping gene and ΔCt ((average of Ct_control_) − Ct_value_). Results are expressed as relative mRNA expression normalized to the gene expression levels of hfMSCs from T25 day 0 and calculated as 2^−ΔCt^.

**Table 1:** **Primer sequences**.

Gene	Forward primer	Reverse primer
B2M	GACTTGTCTTTCAGCAAGGA	ACAAAGTCACATGGTTCACA
ALCAM	ACGATGAGGCAGACGAGATAAGT	CAGCAAGGAGGAGACCAACAA
CD-63	GCCCTTGGAATTGCTTTTGTCG	CATCACCTCGTAGCCACTTCT
f-Actin	GGCATCCTCACCCTGAAGTA	GGTGTGGTGCCAGATTTTC
Runx2	GGAGTGGACGAGGCAAGAGTTT	AGCTTCTGTCTGTGCCTTCTGG
ALP	ACAAGCACTCCCACTTCATC	TTCAGCTCGTACTGCATGTC
ACAN	AGGCAGCGTGATCCTTACC	GGCCTCTCCAGTCTCATTCTC
Sox9	TGGGCAAGCTCTGGAGACTTC	ATCCGGGTGGTCCTTCTTGTG
Col-1	GTCACCCACCGACCAAGAAACC	AAGTCCAGGCTGTCCAGGGATG
Col-2	CGTCCAGATGACCTTCCTACG	TGAGCAGGGCCTTCTTGAG

For gene expression analysis of haMSCs, samples were seeded statically for 3 days followed by another 6 days of static culture or 6 days of dynamic culture in the biaxial bioreactor system or in the perfusion-flow bioreactor. After culture, medium was aspirated from the scaffolds, the samples were transferred to 2 mL Eppendorf tubes and stored at −80°C. Samples for basal gene expression analysis (referred to as day 0 static culture) were retrieved from 2D culture in T25 tissue culture asks (NUNC, Fisher Scientific, the Netherlands) by adding 500 μL of TRIzol^®^ (Invitrogen, the Netherlands) upon reaching 70% confluence. RNA isolation, cDNA synthesis, and RT-PCR were carried out as described for hfMSCs. Results are expressed as relative mRNA expression normalized to the gene expression levels of haMSCs from T25 day 0 and calculated as 2^−ΔCt^.

### Scanning Electron Microscopy Analysis

Cell morphology, attachment, and distribution were characterized by scanning electron microscopy (SEM) analysis with a Philips XL 30 ESEM-FEG (FEI, the Netherlands). Samples were fixed for 30 min in 10% formalin. Subsequently, the samples were dehydrated in sequential ethanol series and critical point dried from liquid carbon dioxide using a Balzers CPD 030 Critical Point Dryer (Leica, Germany). The constructs were gold sputter coated (Cressington, UK) prior to SEM analysis. SEM images were obtained under high vacuum with an acceleration voltage of 30 kV and a working distance of 10 mm.

### Methylene Blue Staining

Cell morphology, attachment, and distribution of haMSCs were qualitatively assessed by methylene blue staining. Samples were fixed for 30 min in 10% formalin, washed with PBS twice, and stained with methylene blue (Sigma, the Netherlands) for 30 s immediately followed by extensive washing with DI-water until the water remained colorless. The samples were imaged using a stereomicroscope (Nikon SMZ800 with Q-imaging Retiga 1300 camera, Nikon Instruments Europe, the Netherlands).

### Histological Analysis

Samples were fixed after live/dead imaging or directly after culture for 30 min in 10% formalin and stored in PBS at 5°C until further processing. Samples were dehydrated using a sequential ethanol series (60, 70, 80, 90, 96, and 100% ethanol, 30 min for each step), and subsequently embedded in glycol methacrylate (GMA). The obtained blocks were sectioned at 5 μm intervals, and stained with hematoxylin and eosin (H&E, Sigma, the Netherlands) for visualization of the nuclei and cytoplasm, and Masson Trichrome (Merck, Germany) to stain for collagen-like ECM formation.

### Statistical Analyses

Results are presented as mean ± SD, and compared using either one-way ANOVA (multiple conditions) with a Bonferroni posttest or Student’s *t*-test (two conditions). Statistical significance was set to *p*-value <0.05 (*).

## Results

### Viability, Distribution, and Gene Expression Profile of hfMSCs

**Figure 2:**
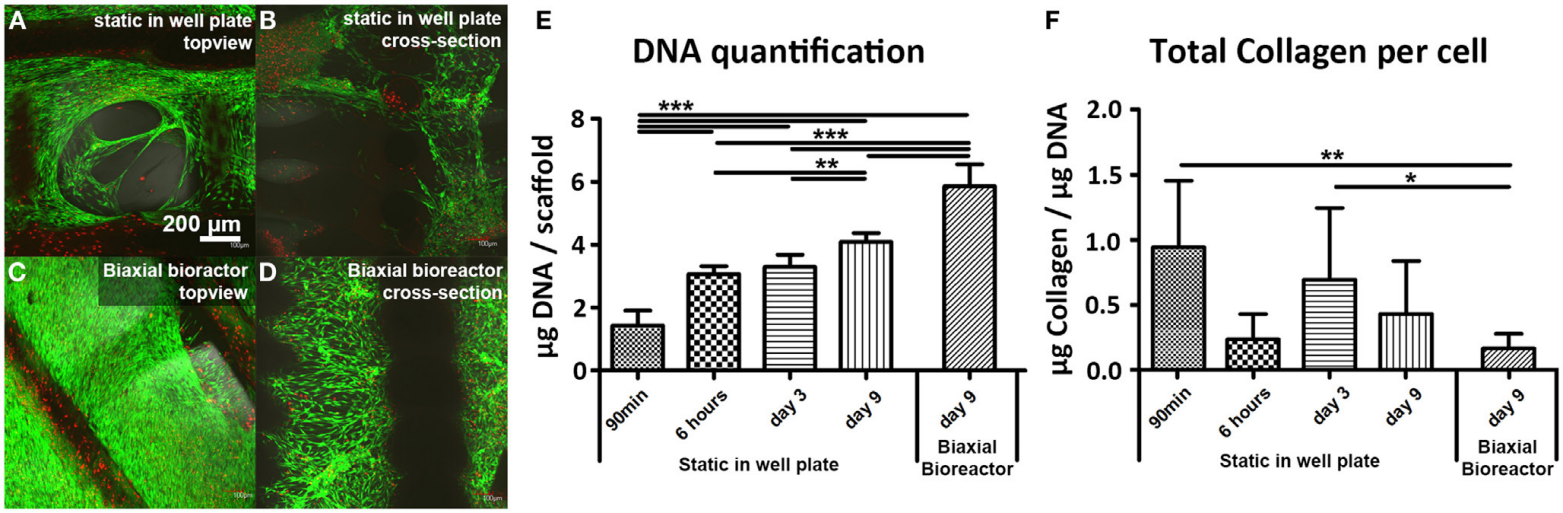
**Live/dead staining showed a high cell viability of fetal hMSCs after 9 days of static culture in a well plate (A,B) and after 3 days of culture in static condition in a plate followed by 6 days of culture in a biaxial bioreactor (C,D)**. Higher numbers of cells were found in the scaffolds after culture in the biaxial bioreactor compared to the scaffolds from static culture. This was confirmed by DNA quantification **(E)** where a significant difference in cell number was found between static culture in a plate and culture in the biaxial bioreactor. DNA quantification also shows a significance increase in cell number in time (90 min, 3, 9 days) when the cells are cultured statically in a well plate. **(F)** Collagen production per cell did not increase in time and significantly decreased upon culture in the biaxial bioreactor compared to 1 day of static culture subsequent to 90 min of seeding in a concentrated suspension and to 3 days of static culture [90 min and 6 h (*n* = 5), day 3 (*n* = 10), day 9 static (*n* = 12), day 9 biaxial bioreactor (*n* = 14), *p < 0.05, **p < 0.01, ***p < 0.001].

Scaffolds seeded with hfMSCs showed comparable viability after 9 days of culture in both well plate (static) and in the biaxial bioreactor (Figures 2A,C, respectively). A high viability was also found 6 h after seeding (Figures S1A,B in Supplementary Material) and after 3 days of static culture (Figures S1C,D in Supplementary Material). In the cross-sectional view of the scaffolds, it could be observed that the number of hfMSCs and their distribution seemed to be enhanced when the constructs were cultured in the biaxial bioreactor (Figure 2D) compared to the scaffolds with hfMSCs cultured statically in a well plate (Figure 2B). Also, the morphology of the cells in the pores of the scaffolds appeared more spread after culture in the biaxial bioreactor (Figures S1E–H in Supplementary Material). The difference in cell number was confirmed by DNA quantification (Figure 2E). Both the scaffolds in static culture and in the biaxial bioreactor showed an increase in cell number over time; yet, the increase in the biaxial bioreactor was significantly higher than in static culture.

Furthermore, allowing the cells to adhere to the scaffold initially for 6 h before adding medium resulted in a significant higher cell seeding efficiency than when the medium was added already after 1.5 h of incubation. A hydroxyproline assay was performed to determine the total amount of intra and extracellular collagen, which is a measure for cells activity and ECM formation (Figure 2F). A small increase in total collagen production per cell was observed between 6 h and 3 days of static culture, whereas the DNA content remained similar. At later timepoints, the DNA content increased, whereas the total collagen content per scaffold remained constant for these conditions, resulting in a decrease of collagen production per cell. This suggests that hfMSCs were mainly proliferating.

**Figure 3:**
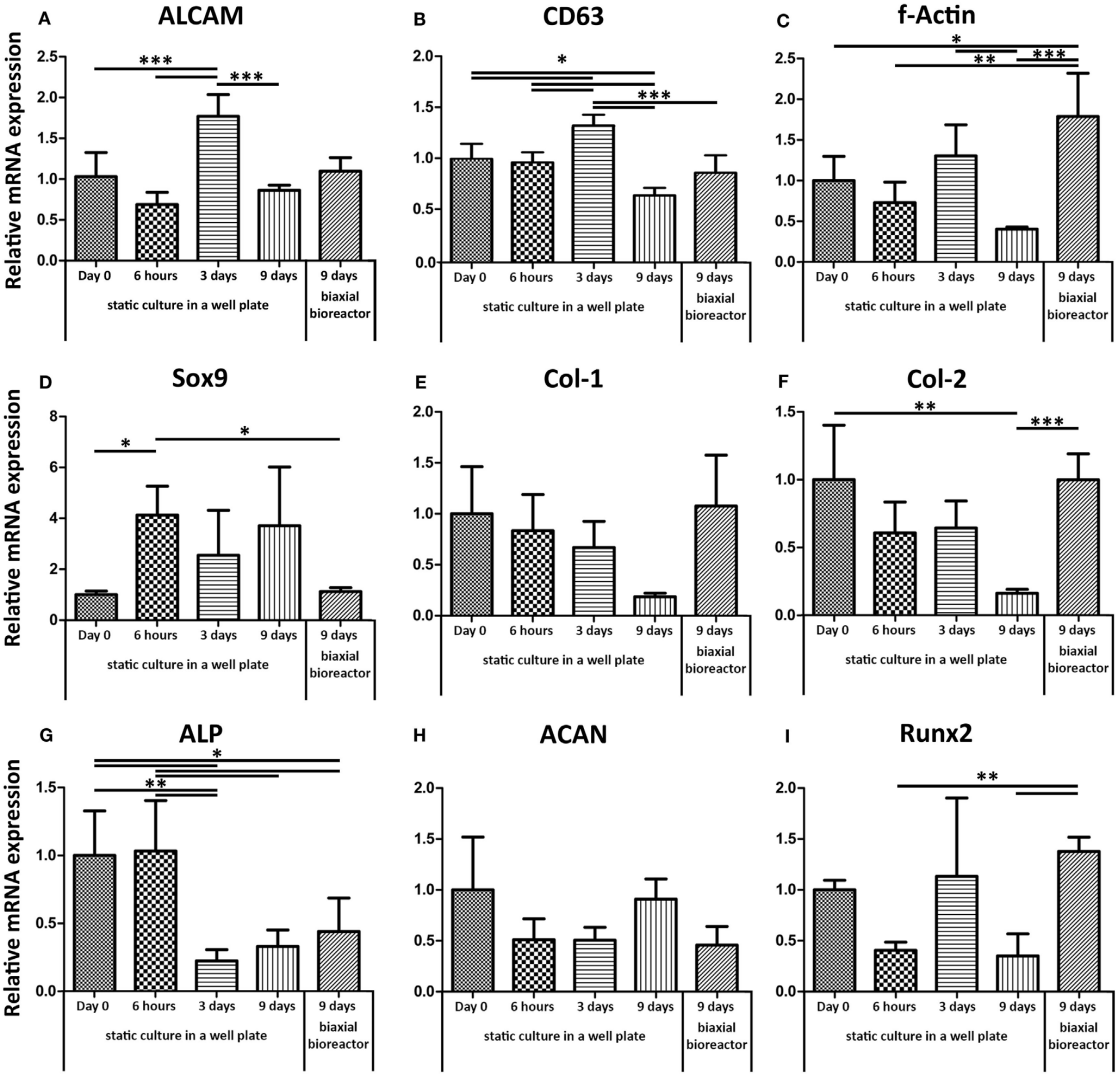
**Relative mRNA expression levels were assessed by qPCR**. The basal mRNA expression levels were determined for hfMSCs from monolayer culture on tissue culture-treated polystyrene (Day 0). **(A,B)** ALCAM and CD63, both genes associated with the maintenance of stemness, did not show significant differences between the basal gene expression levels and the expression levels after 9 days of static or biaxial bioreactor culture. **(C)** F-Actin showed a slight down-regulation after 9 days in static culture and a significant up-regulation after 3 days static followed by 6 days biaxial bioreactor culture. This same trend was found for Runx2 **(I)**. **(G)** ALP was down-regulated for both static and biaxial bioreactor culture. **(H,D)** ACAN and Sox9 were down-regulated in convection culture. **(E,F)** Col-1 and col-2, markers for ECM production, were down-regulated in time in static culture, but retained basal expression levels when the scaffolds were placed in biaxial bioreactor culture [*n* = 4 except for day 9 dynamic (*n* = 5), *p < 0.05, **p < 0.01, ***p < 0.001].

Gene expression levels of ALCAM (CD166, activated leukocyte cell adhesion molecule) and CD63, both indicators for stemness (Jeannet et al., 2013) (Figures 3A,B respectively), fluctuated over time in static culture. ALCAM and CD63 were similarly expressed after 9 days in both static cultures in a well plate and after 9 days of culture in the biaxial bioreactor compared to their basal level measured in cells trypsinized from monolayer culture at a confluence of approximately 70% (day 0 static). F-actin mRNA expression showed a significant twofold up-regulation in dynamic culture and a twofold down-regulation in static culture after 9 days. A similar but less robust trend was found for Runt-related transcription factor 2 (Runx2), associated with early osteogenic differentiation. Alkaline phosphatase (ALP) expression, involved in osteogenesis, was down-regulated over time both for static and bioreactor culture.

Aggrecan (ACAN), a marker for chondrogenesis, did not show any significant differences between any of the conditions. Yet, Sox9, an early marker for chondrogenesis, was up-regulated in static culture in a well plate, whereas the biaxial bioreactor system restored the mRNA expression levels similar to the basal levels (day 0 static culture). Collagen type-1 (col-1) and collagen type-2 (col-2) markers for ECM proteins, abundantly present in bone and cartilage, respectively, were both down-regulated over time in static culture, whereas their expression levels were restored upon culture in the biaxial bioreactor system.

**Figure 4:**
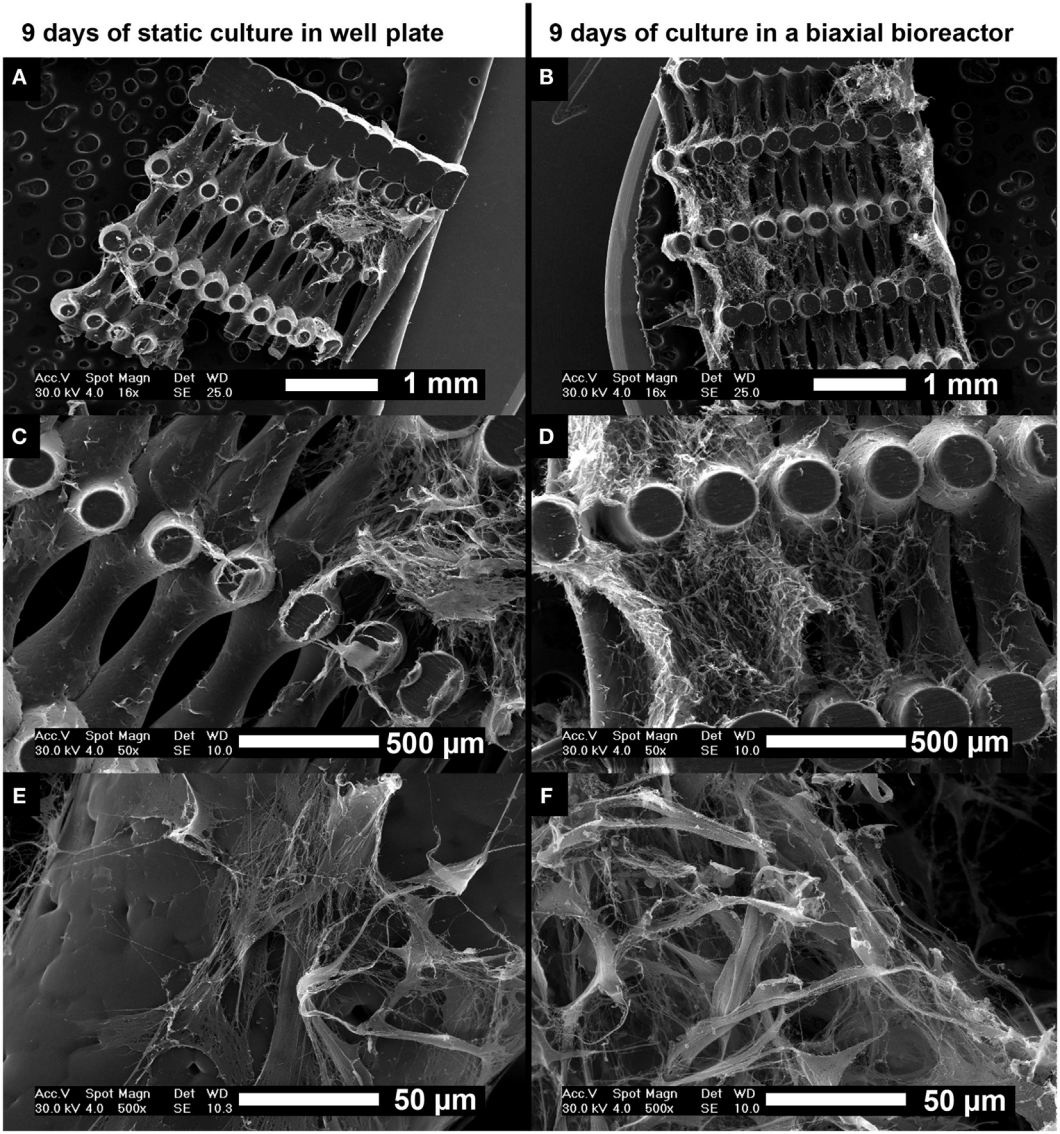
**Scanning electron microscopy images of the distribution of hfMSCs throughout the scaffolds after 9 days of static culture in a well plate (A,C,E) and after 3 days of static followed by 6 days of dynamic culture in a biaxial rotating bioreactor (B,D,F)**. A slightly more homogeneous distribution of cells and ECM was found after culture in the biaxial bioreactor compared to static culture in a well plate. Scale bars represent **(A,B)** 1 mm, **(C,D)** 500 μm, and **(E,F)** 50 μm.

Scanning electron microscopy analysis was performed on cross-sections of the scaffolds after 9 days of culture to assess cell distribution, morphology, and tissue formation (Figure 4). Statically cultured scaffolds (Figures 4A,C,E) showed lower cell numbers compared to scaffolds cultured in the biaxial bioreactor (Figures 4B,D,F). The cellular distribution appeared more homogeneous and the cells invaded the longitudinal pores of the scaffold more profoundly after culture in the biaxial bioreactor system compared to culture in static conditions. There were no large differences observed in ECM formation and cell morphology between static culture and culture in the biaxial bioreactor system. In both conditions, the cells were spread while partly attached to the scaffold material or the secreted ECM. The formed tissue appeared relatively open inside the pores of the scaffolds, whereas the outer layer of tissue found at the bottom or top of the scaffold appeared as a more dense and closed layer.

From the SEM analysis after 6 h (Figures S2A–C in Supplementary Material) and 3 days of static culture (Figures S2D–F in Supplementary Material), it could be seen that the cell morphology changed over time from a rounded shape to a more spread morphology. Similar cell-shape changes were observed in 2D culture where hfMSCs showed a more spread morphology upon attachment to the substrate material. This change in cellular morphology could also be observed in histological analysis by H&E staining (Figure S3 in Supplementary Material). Further analysis with Masson Trichrome staining showed collagen-like materials stained green, which did not show any differences between 9 days of static culture in a well plate and 9 days of culture in the biaxial bioreactor (Figure S4 in Supplementary Material).

### The Cell Adherence, Distribution, and Gene Expression Profile of haMSCs

Comparing the methylene blue staining of haMSCs after 3 days of static culture followed by 6 days of culture in a biaxial bioreactor (Figure S5B in Supplementary Material) to the staining after 9 days of static culture in a well plate (Figure S5A in Supplementary Material), no improvement on cell number and cellular distribution was found upon bioreactor culture. In static culture, haMSCs adhered to the fibers and partly filled the pores of the scaffold probably upon proliferation and ECM production (Figure S5A in Supplementary Material). In the biaxial bioreactor culture, the cells did not fill the pores of the scaffold to the same extend as the cells in static culture. From the cross-sectional view, it could be observed that the distribution of the cells throughout the scaffold was still limited in both conditions. Donor 2 showed limited cell adherence after 3 days of static culture in a well plate followed by 6 days of culture in the perfusion bioreactor (Figure S5C in Supplementary Material).

The DNA content was quantified for two donors in triplicate (Figure S6 in Supplementary Material). The results showed on average a relatively low DNA content (about a threefold less compared to hfMSCs) in both bioreactors. For donor 1, a slight increase in DNA content was found upon bioreactor culture compared to static culture. Donor 2, however, showed a significant difference between static and perfusion culture, and between the biaxial bioreactor and the perfusion bioreactor culture. For this same donor, higher cell numbers were also observed in static culture compared to culture in the biaxial bioreactor or in the perfusion bioreactor when the constructs were cultured statically in a well plate for 31 days prior to a 14 days culture in the two bioreactor systems (Figure S7 in Supplementary Material). These findings were confirmed by DNA assay in which a significant drop in DNA content was observed after the scaffolds were cultured in the biaxial bioreactor or perfusion bioreactor culture compared to the DNA content after 31 and 45 days of static culture (Figure S8 in Supplementary Material).

**Figure 5:**
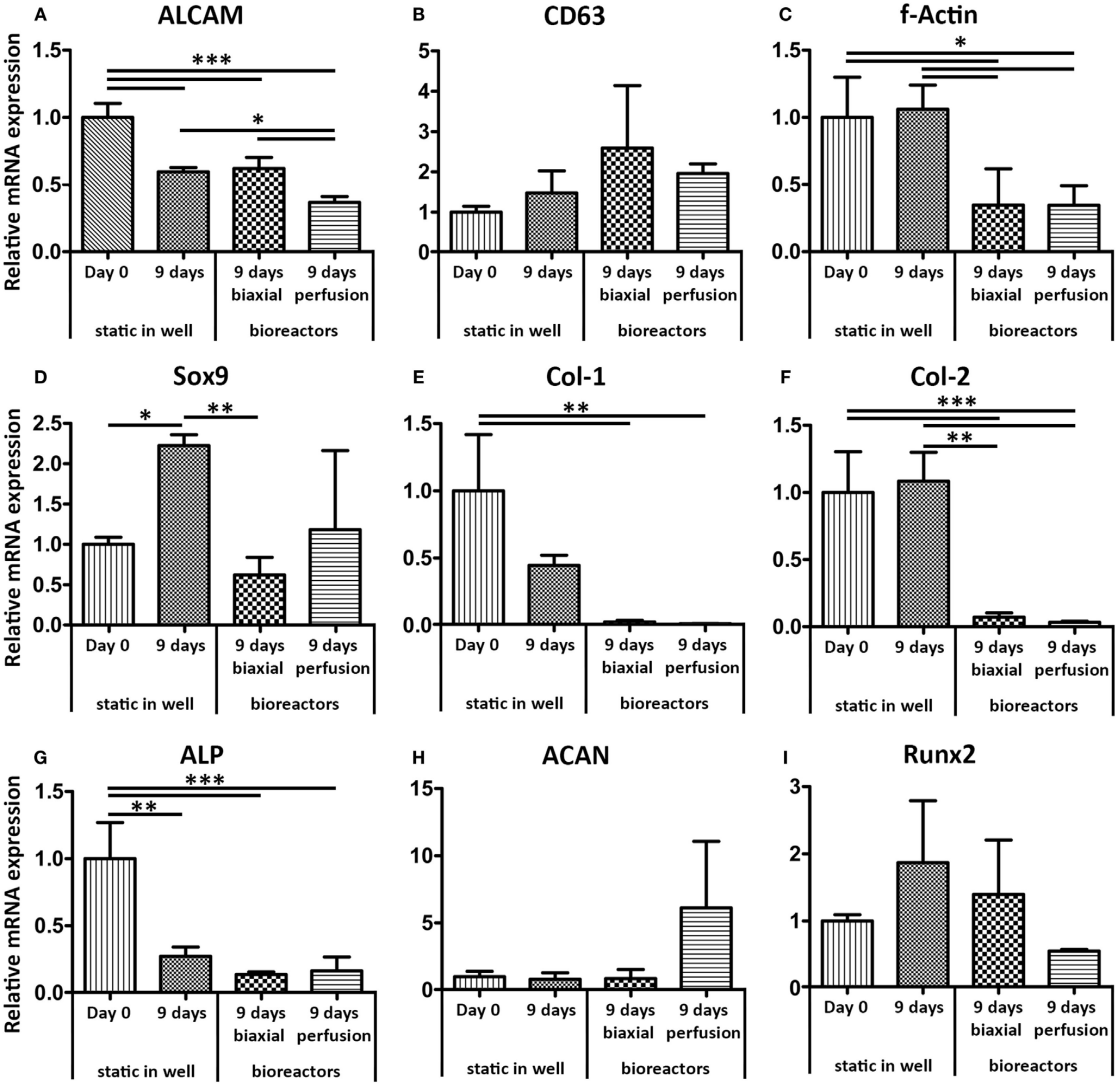
**Relative mRNA expression levels of haMSCs in static, biaxial bioreactor, and perfusion bioreactor culture were assessed by qPCR**. **(A)** ALCAM showed a twofold significant down-regulation after 9 days of perfusion culture compared to static and biaxial bioreactor cultures. **(B)** CD63 showed no significant differences between the different culture systems. **(C)** F-Actin showed a down-regulation after 9 days in both dynamic culture systems compared to static culture. (**D**) Sox9 was significantly up-regulated in static culture in a well plate, whereas the bioreactor systems restored the mRNA expression levels similar to the basal levels (day 0 static culture). **(E,F)** Col-1 and Col-2, markers for extra-cellular matrix production, were down-regulated in both dynamic culture systems. **(G)** ALP showed a significant down-regulation in all conditions after 9 days of culture compared to day 0 in static culture. **(H)** ACAN showed a 10-fold up-regulation after perfusion culture compared to static and biaxial bioreactor culture; however, this change was not statistically significant. **(I)** For Runx2, no significant differences between the different culture conditions were found (*n* = 3, *p < 0.05, **p < 0.01, ***p < 0.001).

The gene expression profile of haMSCs was assessed for the same genes as hfMSCs (Figure 5). The gene expression levels were determined after 9 days of static culture and compared to gene expression levels of haMSCs after 3 days of static culture followed by 6 days of dynamic culture in the biaxial bioreactor or in the perfusion bioreactor. A significant twofold down-regulation of ALCAM was found for haMSCs cultured in a perfusion bioreactor compared to static culture and culture in the biaxial bioreactor. F-Actin, col-1, and col-2 showed a significant down-regulation in both dynamic culture systems compared to static culture. Sox-9 was significantly down-regulated in the biaxial bioreactor and ACAN was up-regulated a 10-fold in perfusion culture compared to static culture. However, this change was not statistically significant.

## Discussion

Mesenchymal stromal cells derived from bone marrow have been used as a cellular source in several tissue engineering applications due to their availability, ease of isolation from autologous source and therewith reduced immunological-related risks upon re-implantation. Moreover, MSCs have shown a certain degree of plasticity by the ability to differentiate and transdifferentiate after differentiation into several well-defined cell lineages both *in vitro* and *in vivo* (Phinney et al., 1999; Grove et al., 2004; Song and Tuan, 2004; Phinney, 2007; Russell et al., 2010; Ullah et al., 2013).

The multipotency of MSCs is often assessed by soluble factor-induced differentiation in which the results of histological or biochemical assays are used to characterize cell fate. However, instead of focusing on changes in MSCs phenotype by quantifying the expression of differentiation-related genes, it could be also of interest to assess to what extent the cellular phenotype of MSCs can be maintained by investigating genes related to multipotency. In the past decades, researchers focused on defining stemness and identifying stemness markers to be able to more directly assess the potency of patient-derived MSC populations (Menicanin et al., 2009).

A known drawback in the use of haMSC is their slow proliferation time and generally limited proliferation capacity. More recently, hfMSCs have been characterized showing a higher proliferation and osteogenic differentiation capacity and reduced immunogenicity when compared to mesenchymal stem cell populations derived from the umbilical cord, adult adipose tissue, or adult bone marrow in 3D culture systems both *in vitro* and *in vivo* (Zhang et al., 2009a). Here, we have assessed hfMSCs and haMSCs behavior in 3D scaffolds with respect to cell attachment, proliferation, and genetic profile.

Static and bioreactor culture conditions both resulted in similar cell viability on the PEOT/PBT scaffolds (Figures 2A–D). However, the number of hfMSCs in the biaxial bioreactor culture was higher (Figure 2E) and the distribution of the cells throughout the scaffolds seemed to be improved with respect to the scaffolds in static culture in a well plate (Figures 2A–D and Figure 4). With respect to the gene expression levels, it could be concluded that 6 days of biaxial bioreactor culture subsequent to 3 days of static culture restored the basal gene expression levels, found at day 0 in static monolayer culture, for all genes except ALP and f-actin. Similar results were found by a study of Katayama et al. (2013), in which no change in gene expression levels of stem cell markers, such as ALCAM, was found in adult hMSCs cultured on collagen sheets under perfusion flow.

Considering the genes involved in ECM production, such as f-actin, col-1, and col-2, static culture seemed to provide insufficient cues to the cells to activate ECM formation-related pathways. Col-1 and col-2 expressions were maintained in the biaxial bioreactor culture and down-regulated in static culture. This indicated that the hfMSCs in the biaxial bioreactor probably experienced shear forces, due to a sort of convection flow, resulting in activation of the f-actin pathway among others, which could subsequently lead to an increase in tissue formation (Sansores-Garcia et al., 2011). Furthermore, shear forces have been known to induce osteogenic differentiation, which could explain the initial significant decrease of ALP expression in static culture and the subsequent small increase in ALP in the biaxial bioreactor (Figure 3) (Yeatts et al., 2012). On the other hand, a recent study of Kock et al. (2014) has shown a negative influence of perfusion flow on cartilage-like ECM production of chondrogenic pre-differentiated hMSCs, although these changes were not evident from gene expression analysis (Kock et al., 2014).

Scanning electron microscopy analysis (Figures 4E,F) showed an increase in tissue formation and tissue density was observed for dynamically cultured samples compared to statically cultured scaffolds. Although 6 days of culture in the biaxial bioreactor may have altered the gene expression levels compared to static culture, no difference was found on total collagen production by biochemical and histological analysis (Figure 2F and Figures S4E–H in Supplementary Material, respectively). However, the number of cells in the interior of the scaffold in the biaxial bioreactor was higher than in static culture conditions, indicating that bioreactor culture did improve cellular distribution.

Although hfMSCs have shown some beneficial properties over haMSCs in previous studies, the application of haMSCs remained of interest due to their more immediate clinical relevance. Fetal MSCs cannot be used from autologous source and their use involves more ethical issues. Therefore, the use of haMSCs on these scaffolds in the biaxial bioreactor was evaluated as well. A perfusion-flow bioreactor was introduced in parallel to be able to compare the results obtained in the biaxial bioreactor culture to both static and perfusion culture conditions. The cell number of haMSCs per scaffold assessed by DNA assay after 9 days of static culture was found to be significantly lower for both haMSC donors (Figure S6 in Supplementary Material) compared to ­hfMSCs (Figure 2E). Cell seeding efficiency of haMSCs has already shown to be limited and donor dependent in one of our previous studies, in which several seeding parameters were optimized to decrease cell loss after seeding (Leferink et al., 2013).

For the two haMSCs donors, no difference in the number of cells was found upon culture in the biaxial bioreactor compared to static culture conditions. Similar results were found in a study of Stiehler et al. (2009), in which hMSCs seeded on PLGA-based scaffolds did not show any difference in cell number after 7 days of convection culture compared to static culture. In other studies, perfusion culture was applied on porous 3D scaffolds and showed to improve cell proliferation and scaffold colonization (Alvarez-Barreto et al., 2007; Bjerre et al., 2008; Grayson et al., 2008, 2011; Schumacher et al., 2010). In our study, however, perfusion culture showed to significantly decrease the number of haMSCs from donor 2 compared to static culture or culture in the biaxial bioreactor. This could be due to the highly interconnected and organized pore network of additive manufactured scaffolds compared to more conventional sponges and non-woven scaffolds with random fiber organization used in previous studies. A similar negative effect was reported in a study by Bjerre et al. (2011) in which perfusion culture unfavorably changed the morphology and vitality of haMSCs (Bjerre et al., 2011).

Differences in initial cell attachment between the two donors could be an effect of differences in heterogeneity of cell populations due to donor variation (DiGirolamo et al., 1999; Phinney et al., 1999; Siddappa et al., 2007; Leferink et al., 2013; Siegel et al., 2013). Changes in cell response to the introduction of perfusion flow could also be related to several other factors such as the amount of ECM produced at the moment of transfer from static culture to the perfusion-flow system and the distribution of the cells and ECM throughout the scaffold. Upon closure of the longitudinal pores of the scaffold due to the presence of the formed ECM in static culture, the pressure, or the perfusion flow is expected to be increased, which could have led to tissue detachment. We hypothesized that prolonging the static culture period prior to applying bioreactor culture could result in more ECM with stronger binding and entanglement to the scaffold material (Griffon et al., 2011). Therefore, the scaffolds were cultured statically for 31 days followed by 14 days of culture in the biaxial bioreactor or in the perfusion bioreactor. From cross-sectional views on methylene blue stained scaffolds (Figure S7 in Supplementary Material), it could be observed that the cells and tissues indeed seemed to be detached from the scaffold upon dynamic culture, whereas in static conditions, a homogeneous distribution of cells and tissue throughout the scaffold was found. These results were confirmed by DNA quantification, which showed a significant loss of cells upon transferring the constructs from static to bioreactor culture (Figure S8 in Supplementary Material). In both the biaxial bioreactor and the perfusion bioreactor, loosened cell sheets were found in the longitudinal pores of the scaffolds. A difference between the two bioreactor systems was found with respect to the adherence sites of the cells. In perfusion culture conditions, cells were found on the outer layer of scaffold material, whereas in the biaxial bioreactor, lesser cells were found to reside on the outer layer of the scaffold.

The gene expression levels of f-actin, col-1, and col-2 previously showed to be significantly up-regulated in hfMSCs, whereas a significant down-regulation was found in haMSCs when comparing the basal gene expression levels and the levels after 9 days of static culture to the levels after 3 days of static culture followed by 6 days of culture in the biaxial bioreactor. For Sox-9, a similar response was found for hfMSCs compared to haMSCs. ALCAM, CD63, and ACAN did not show any significant differences in hfMSCs and haMSCs cultured statically or dynamically in the biaxial bioreactor. Larger differences in gene expression levels were found between perfusion culture and static culture than between culture in the biaxial bioreactor and static culture. ALCAM, f-Actin, col-1, and col-2 were significantly down-regulated in perfusion culture compared to static culture after 9 days. This could have been related to the lower cell number as a consequence of relatively high shear forces involved in a perfusion culture system.

Overall, the results presented in this study suggested that a more homogeneous cell distribution could be obtained for fetal MSCs compared to adult ones in all conditions, and that the benefit of applying bioreactor culture over static culture was not obvious for adult MSCs. Although the experiments with haMSCs were not as successful as with hfMSCs with respect to cell number and distribution, we believe that there is still the potential for a bioreactor system in a later stage of tissue culture with haMSCs. When static cultures lead to complete filling of the scaffold with closure of the pores, cells in the scaffold interior might undergo necrosis by mass transfer limitations. At that stage, a dynamic system, such as the biaxial rotating vessel presented in this study, might become favorable over static culture. Furthermore, in our study, the complex influence of rotational speed and medium perfusion speed on the fluid dynamics was not modeled. Screening the influence of these parameters on MSC fate in additive manufactured scaffolds might result in optimal culture conditions in which a homogeneous distribution throughout the scaffold can be achieved without compromising cells viability or altering MSCs phenotype.

## Conclusion

The biaxial rotating vessel bioreactor used in this study has shown to improve hfMSCs distribution and proliferation in 3D additive manufactured PEOT/PBT scaffolds. The hfMSCs in the biaxial bioreactor produced ECM while retaining a comparable gene expression profile as the basal levels of hfMSCs seeded statically in 2D. Static culture on 3D scaffolds with hfMSCs showed down-regulation of f-actin, col-1, col-2, ALP, and runx2 gene expression levels and also resulted in less ECM production and lower cell numbers than in the biaxial bioreactor, thus suggesting a more quiescent state of hfMSCs. As a third well-known dynamic culture system, a perfusion bioreactor was introduced for the experiments with a more clinically relevant haMSCs population. No increase in cell number and cellular distribution upon culture in the biaxial or the perfusion bioreactor was observed for haMSCs, even after an extended period of static culture prior to culture in the biaxial bioreactors. In addition, the gene expression profile of haMSCs showed a different response to the culture in the biaxial bioreactor than the profile of hfMSCs. There were no consistent significant differences in cell number between 9 days of static culture and 3 days of static culture followed by 6 days of bioreactor culture in both the biaxial bioreactor and the perfusion-flow bioreactor. Overall, the biaxial rotating vessel bioreactor introduced in this study has shown to maintain ­hfMSCs viability and distribution throughout the scaffold, without inducing differentiation. Therefore, this system could serve as a tool to study cell activity in distinct 3D scaffolds.

## Conflict of Interest Statement

The authors declare that the research was conducted in the absence of any commercial or financial relationships that could be construed as a potential conflict of interest.
