# Supplementary material for: Distribution and Viability of Fetal and Adult Human Bone Marrow Stromal Cells in a Biaxial Rotating Vessel Bioreactor after Seeding on Polymeric 3D Additive Manufactured Scaffolds
Source: Front Bioeng Biotechnol. 2015 Oct 23;3:169. doi: 10.3389/fbioe.2015.00169 (PMC4617101; doi:10.3389/fbioe.2015.00169)
Supplement: Supplementary file 1 [file data_sheet_1.pdf]

Supplementary Material

Distribution and viability of fetal and adult human bone marrow stromal cells in a biaxial rotating vessel bioreactor after seeding on polymeric 3D rapid prototyped scaffolds

Anne M. Leferink[a,c], Yhee-Cheng Chng[b], Clemens A. van Blitterswijk[a,c] and Lorenzo Moroni[a,c]*

[a] *Department of Tissue Regeneration, MIRA institute, University of Twente, Enschede, The Netherlands.* [b] *Quintech Life Sciences Pte Ltd, Singapore* [c] *Department of Complex Tissue Regeneration, Faculty of Health, Medicine and Life Sciences, Maastricht University, Maastricht, The Netherlands*

* **Corresponding author:** Lorenzo Moroni, Department of Complex Tissue Regeneration, MERLN institute, Maastricht University, Universiteitssingel 40, 6229ER Maastricht, The Netherlands, *E-mail address*: l.moroni@maastrichtuniversity.nl

1 Supplementary Figures and Tables

1.1 Supplementary Figures

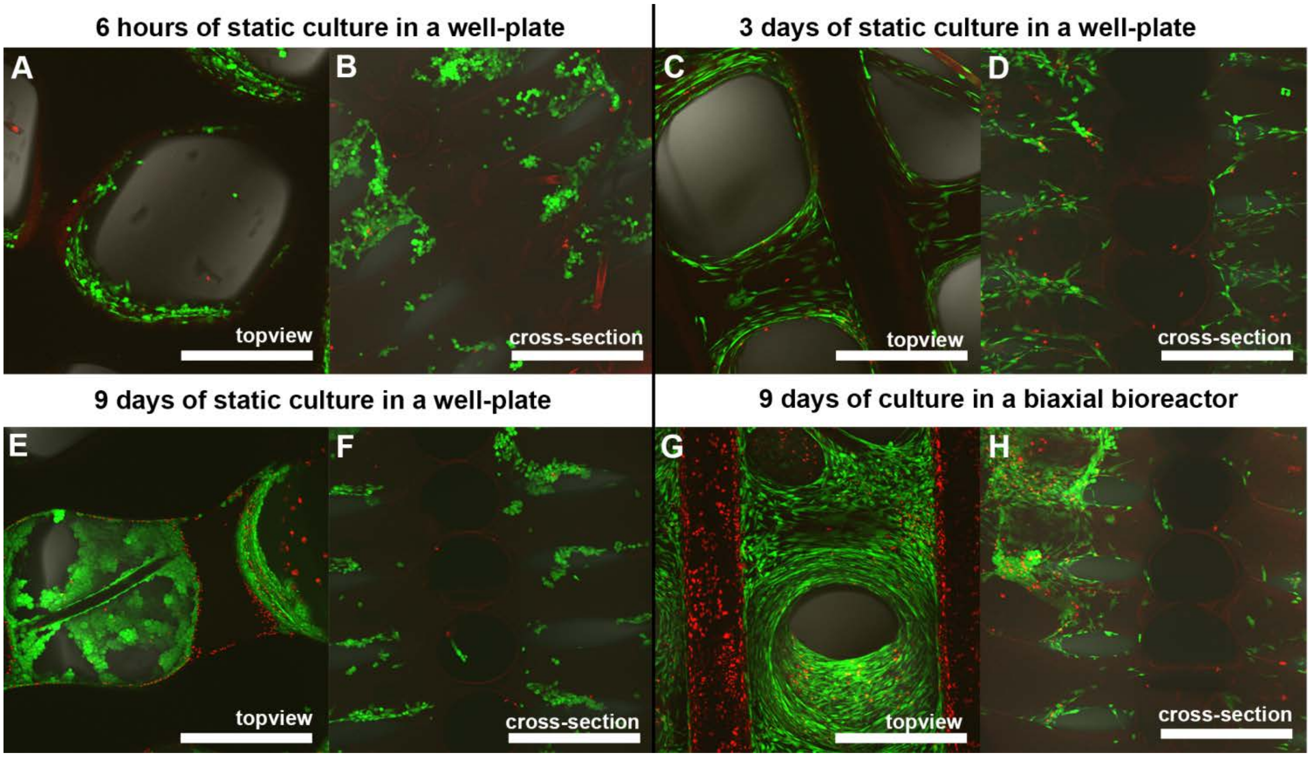

Supplementary Figure 1. Live/dead staining of hfMSCs after 6 hours (A, B), 3 days (C, D) and 9 days (E, F) of static culture and after 3 days of static followed by 6 days of culture in a biaxial rotating bioreactor (G, H). In both static and bioreactor culture, cells showed high cell viability and an improved distribution with respect to earlier time-points. However, it seemed that in the biaxial bioreactor hfMSCs were present in higher numbers. Scale bars represent 500 µm.

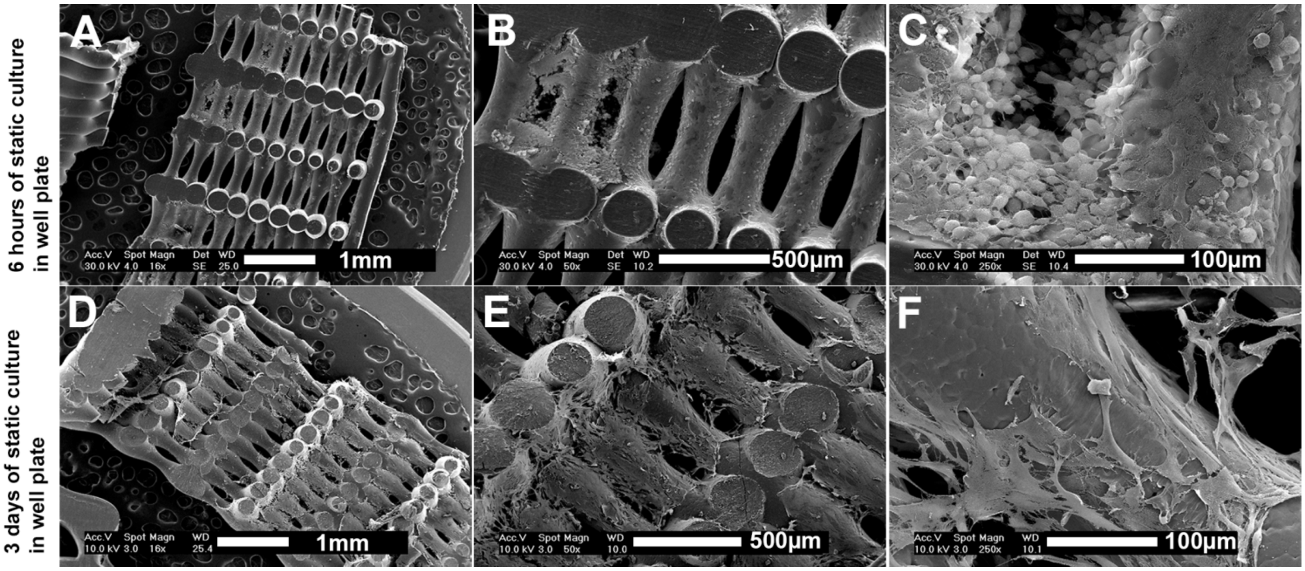

Supplementary Figure 2. Scanning electron microscopy images of hfMSCs 6 hours (A-C) and 3 days (E-F) after seeding and culturing in static condition in a well-plate. Scale bars represent (A, D) 1mm, (B, E) 500 µm, (C, F) 100 µm

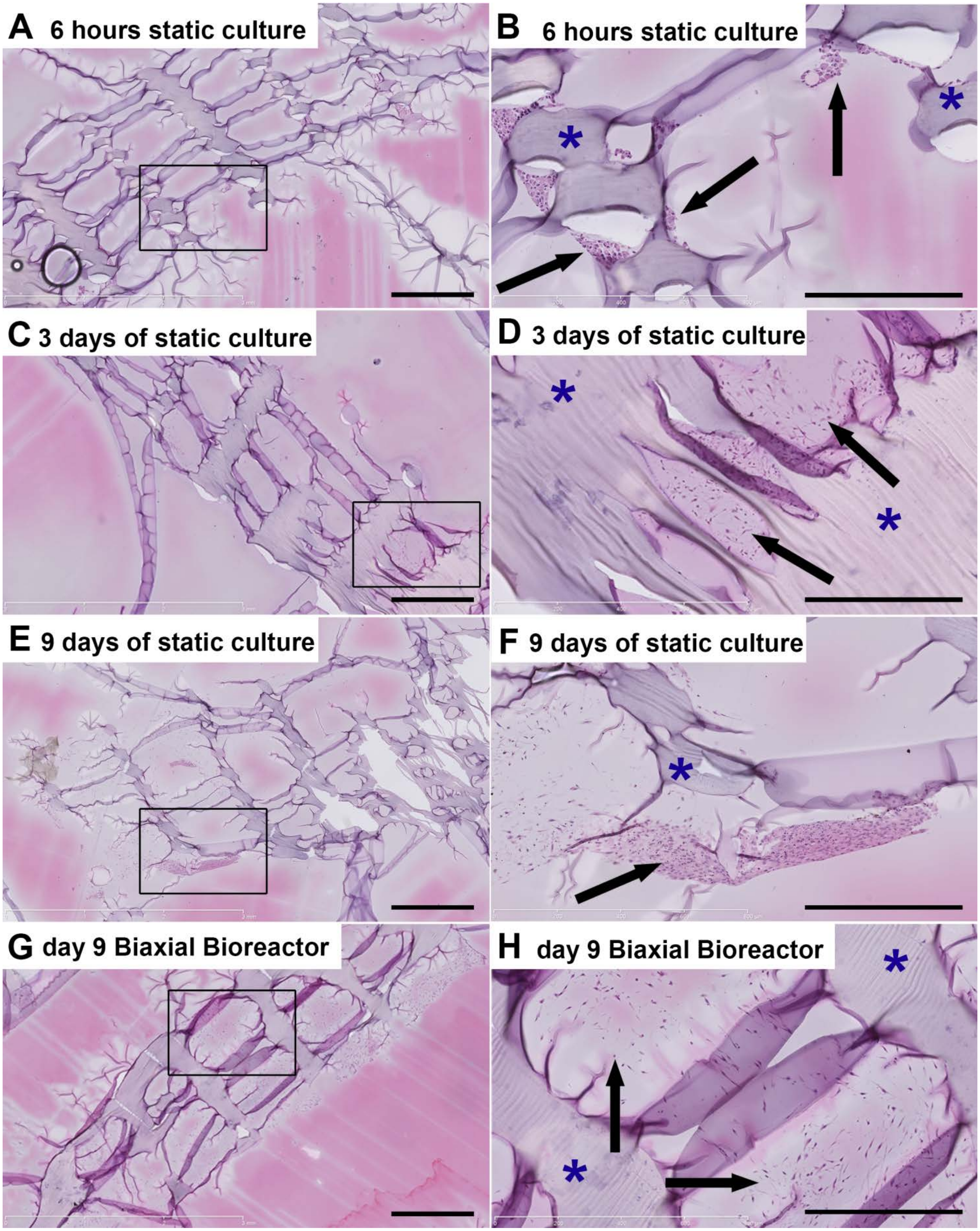

Supplementary Figure 3. Hematoxylin and Eosin staining were applied to assess hfMSCs distribution throughout the scaffold after 6 hours (A, B), 3 days (C, D) and 9 days (E, F) of static

culture and after 3 days of static followed by 6 days of culture in a biaxial rotating bioreactor (G, H). Arrows indicate cells, asterisks (*) indicate scaffold material. Scale bars represent (A, C, E, G) 1mm, (B, D, F, H) 500 µm.

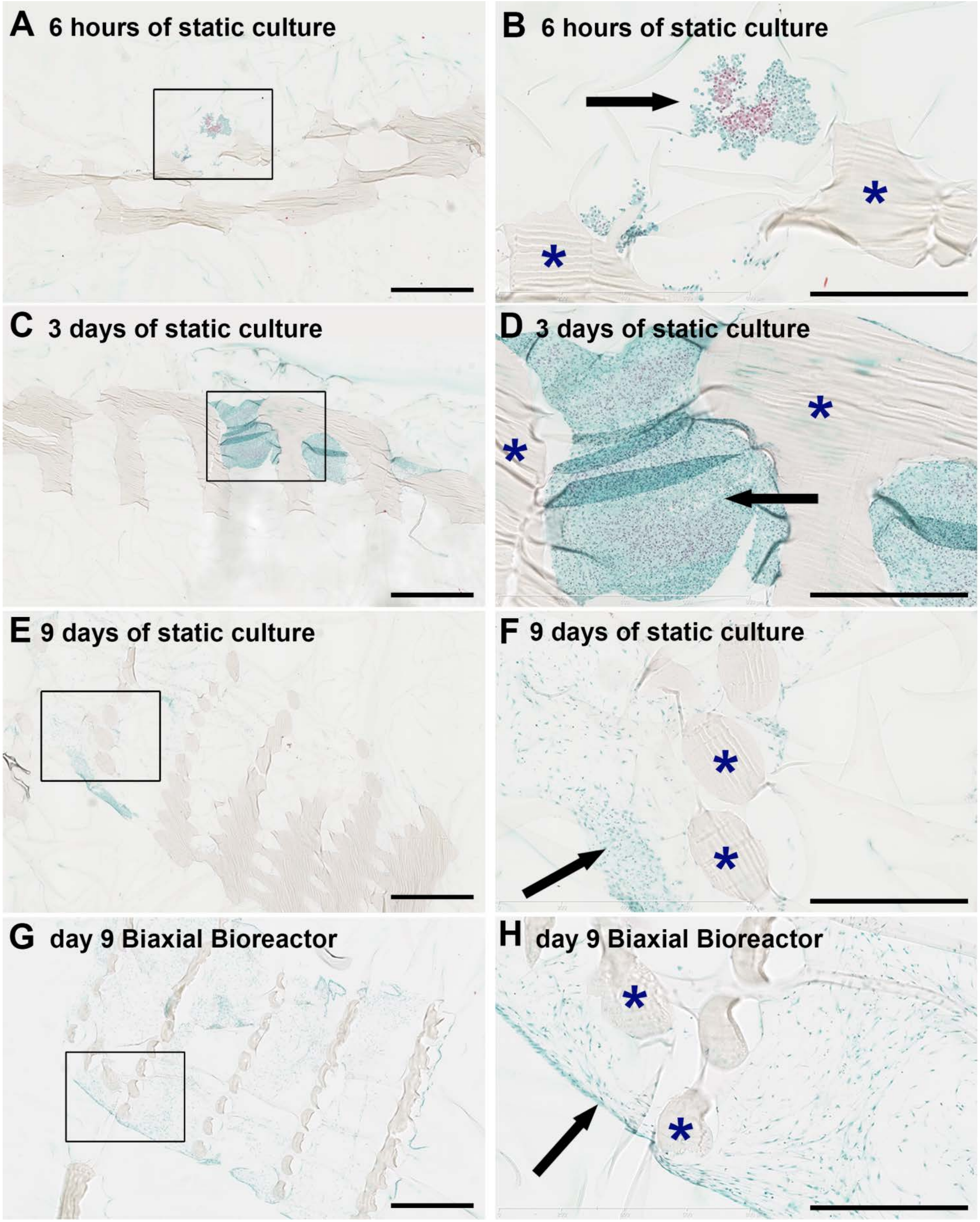

Supplementary Figure 4. Masson Trichrome staining of collagen-like proteins (green) and hfMSCs (green/dark brown) after 6 hours (A, B, top-view), 3 days (C, D, top-view) and 9 days (E, F, cross-section) of static culture and after 3 days of static followed by 6 days of culture in a biaxial rotating bioreactor (G, H, cross-sectional). Arrows indicate cells, asterisks (*) indicate scaffold material. Scale bars represent (A, C, E, G) 1mm, (B, D, F, H) 500 µm.

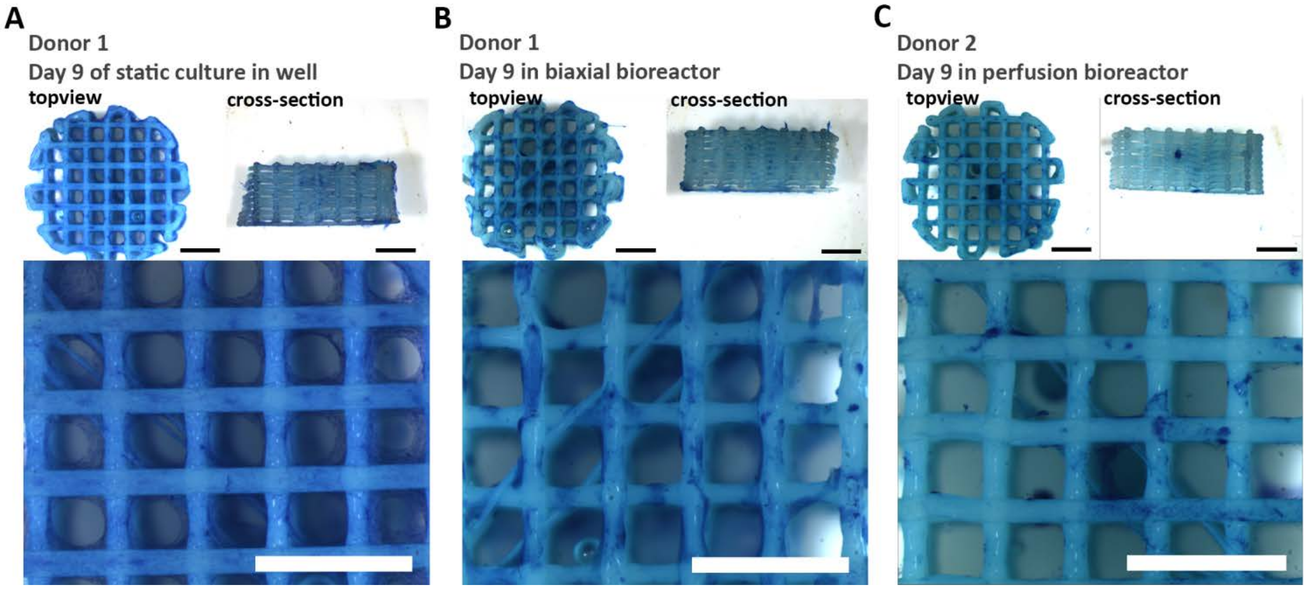

Supplementary Figure 5. Methylene Blue staining of haMSCs of donor 1 after (A) 9 days of static culture in a well plate and after (B) 3 days static culture in a well plate followed by 6 days of culture in a biaxial bioreactor system. (C) Methylene Blue staining of haMSCs of donor 2 after 3 days of static in a well-plate followed by 6 days of culture in a perfusion bioreactor system. Scale bars represent 2 mm.

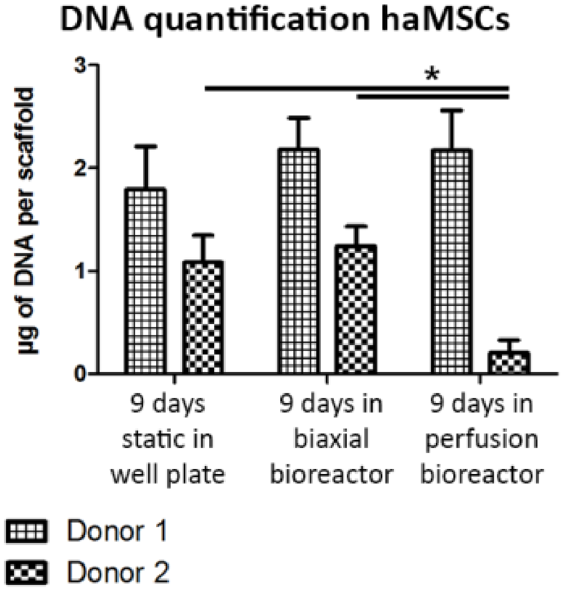

Supplementary Figure 6 DNA quantification of haMSCs after 9 days of culture on scaffolds in a well plate or after 3 days of static culture followed by 6 days of culture in a bioreactor. (n=3, * $p<0.05$)

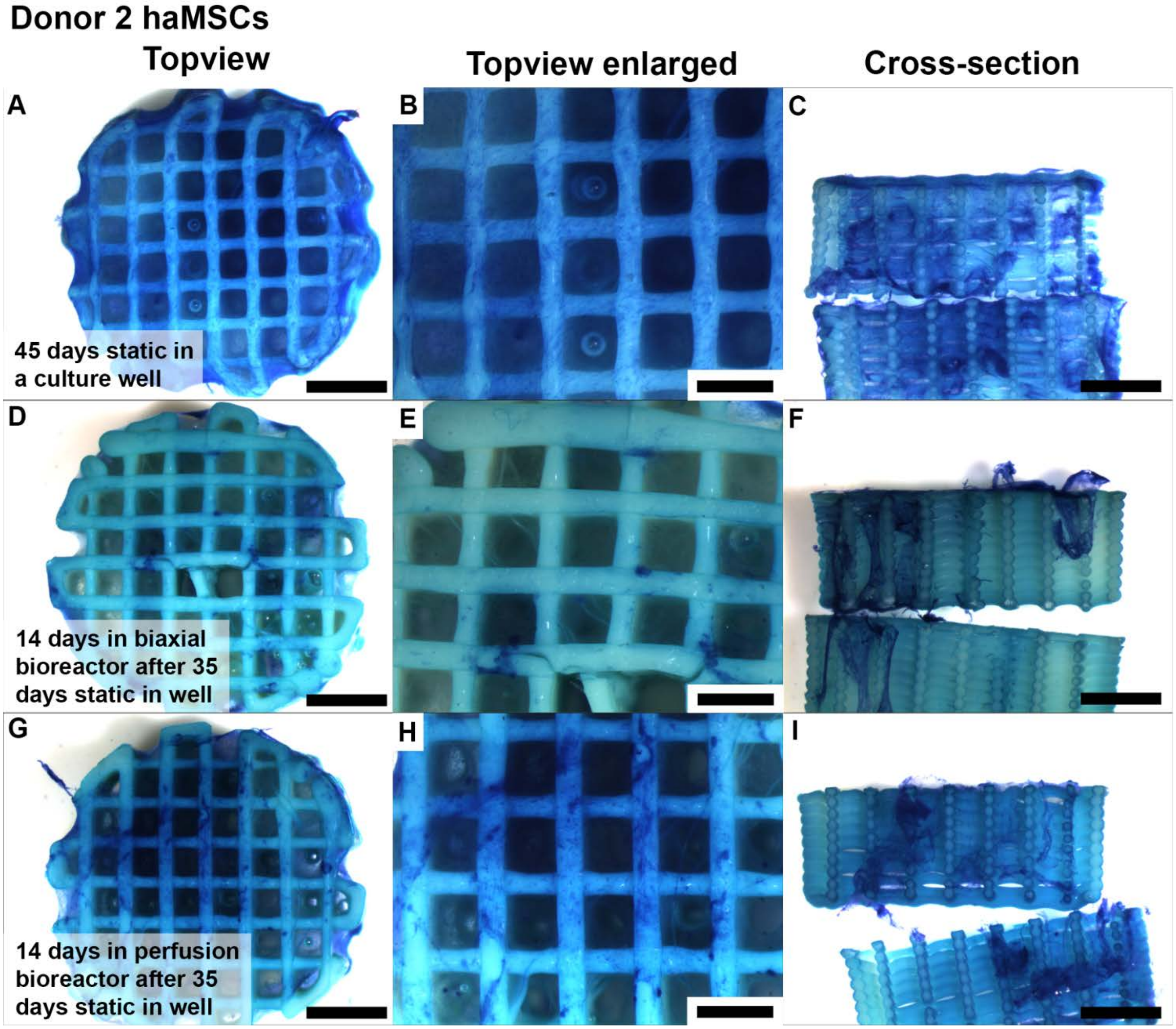

Supplementary Figure 7. Methylene Blue staining of donor 2 haMSCs after 31 days of static culture followed by another 14 days of static culture(A-C), 14 days of culture in a biaxial rotating bioreactor (D-F) or 14 days of culture in a perfusion bioreactor (G-I). The highest number of cells with the most homogeneous cell distribution was found after static culture. It can be seen that the cell and tissue matrix was disturbed under the influence of bioreactor culture. In the cross-sectional view of the scaffolds (C, F, I) it can be seen that in the bioreactors cell sheets were released from the scaffold whereas in static culture in a well plate tissue and cells were maintained in the longitudinal pores of the scaffold. Scale bars represent (A, C, D, F, G, I) 2 mm and (B, E, H) 1 mm.

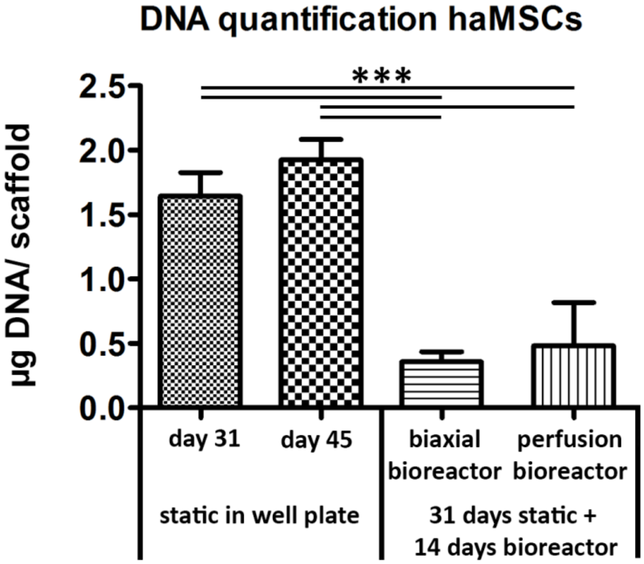

Supplementary Figure 8. DNA quantification of donor 2 haMSCs after 31 days of static culture (day 31) and after 31 days of static culture followed by another 14 days of static culture (day 45), 14 days of culture in the biaxial bioreactor or in the perfusion culture bioreactor. (n=3, * p<0.05, ** p<0.01, *** p<0.001.
